# Fish Oil-Derived Long-Chain n-3 Polyunsaturated Fatty Acids Reduce Expression of M1-Associated Macrophage Markers in an *ex vivo* Adipose Tissue Culture Model, in Part through Adiponectin

**DOI:** 10.3389/fnut.2015.00031

**Published:** 2015-10-13

**Authors:** Anna A. De Boer, Jennifer M. Monk, Danyelle M. Liddle, Krista A. Power, David W. L. Ma, Lindsay E. Robinson

**Affiliations:** ^1^Department of Human Health and Nutritional Sciences, University of Guelph, Guelph, ON, Canada; ^2^Guelph Food Research Centre, Agriculture and Agri-Food Canada, Guelph, ON, Canada

**Keywords:** obesity, adipose tissue, M1 macrophage, n-3 polyunsaturated fatty acids, adiponectin, lipopolysaccharide

## Abstract

Adipose tissue (AT) macrophages (ATM) play a key role in obesity-associated pathologies, and their phenotype can be influenced by the local tissue microenvironment. Interestingly, long-chain n-3 polyunsaturated fatty acids (LC n-3 PUFA) and the LC n-3 PUFA-upregulated adipokine, adiponectin (Ad), may mitigate excessive ATM inflammatory M1-polarization responses. However, to what extent LC n-3 PUFA and Ad work in concert to affect macrophage phenotype has not been examined. Thus, we used an established *ex vivo* AT organ culture model using visceral AT from mice fed a control (CON; 10% w/w safflower oil) n-6 PUFA-rich diet or an isocaloric fish oil (FO; 3% w/w menhaden oil + 7% w/w safflower oil)-derived LC n-3 PUFA-rich diet to generate AT conditioned media (ACM). We then evaluated if CON or FO ACM affected macrophage polarization markers in a model designed to mimic acute [18 h ACM plus lipopolysaccharide (LPS) for the last 6 h] or chronic (macrophages treated with LPS-challenged CON or FO ACM for 24 h) inflammation ± Ad-neutralizing antibody and the LPS-neutralizing agent, polymyxin B. In the acute inflammation model, macrophages treated with FO ACM had decreased lipid uptake and mRNA expression of M1 markers (*Nos2*, *Nf*κ*b*, *Il6*, *Il18*, *Ccl2*, and *Ccl5*) compared with CON ACM (*p* ≤ 0.05); however, these effects were largely attenuated when Ad was neutralized (*p* > 0.05). Furthermore, in the chronic inflammation model, macrophages treated with FO ACM had decreased mRNA expression of M1 markers (*Nos2*, *Tnf*α, *Ccl2*, and *Il1*β) and IL-6 and CCL2 secretion (*p* ≤ 0.05); however, some of these effects were lost when Ad was neutralized, and were further exacerbated when both Ad and LPS were neutralized. Taken together, this work shows that LC n-3 PUFA and Ad work in concert to suppress certain M1 macrophage responses. Thus, future strategies to modulate the ATM phenotype should consider the role of both LC n-3 PUFA and Ad in mitigating obese AT inflammation.

## Introduction

In obesity, adipose tissue macrophages (ATM) play a key role in adipose tissue (AT) inflammation and subsequent development of obesity-associated pathologies, such as local and systemic insulin resistance (1, 2). More specifically, circulating monocytes accumulate in obese AT, particularly in visceral depots, wherein they differentiate into ATM to help with tissue remodeling and lipid homeostasis (3–5). Importantly, paracrine interactions, or cross-talk, between adipocytes and ATM play a key role in determining macrophage polarization status (i.e. M1 or M2) and the resultant AT secretory profile. Many ATM exhibit an inflammatory M1 phenotype, characterized by increased lipid content, NLRP3 inflammasome activation, expression of the integrin (CD11b) and high levels of anti-microbicidal iNOS (murine only), antigen presentation via MHCII, and secretion of inflammatory cytokines, such as TNFα and IL-6 (6–9). Mechanistically, TNFα and IL-6 feedback onto adipocytes through paracrine signaling to sustain secretion of adipocyte-derived inflammatory mediators (e.g., CCL2, IL-6) and release of fatty acids through lipolysis (10). Finally, some ATM exhibit a less inflammatory M2 phenotype, characterized by surface expression of scavenging receptors, such as CD206, antigen presentation, and secretion of the anti-inflammatory cytokine, IL-10 (6).

Obesity is also characterized by increased circulating levels of gut bacteria-derived lipopolysaccharide (LPS), a condition termed as metabolic endotoxemia (11), which leads to LPS accumulation in AT (4). While some macrophages can exhibit endotoxin tolerance or hypo-responsiveness when re-stimulated with LPS (12), it is unclear if this occurs in macrophages within obese AT. Interestingly, both the full-length (13–15) and globular (16–18) isoforms of the anti-inflammatory adipokine, adiponectin (Ad), have been shown to promote a response similar to endotoxin tolerance and macrophage polarization toward the M2 phenotype, or enhance M2 polarization *in vitro* (19) and *in vivo* (20). Therefore, Ad could potentially serve to re-direct the cyclic inflammatory cross-talk between adipocytes and M1 macrophages in obesity.

Given the impact of the ATM phenotype on subsequent inflammatory processes and AT dysfunction, strategies to alter ATM polarization status may be useful in mitigating obesity-related inflammation. Of interest, dietary long-chain polyunsaturated fatty acids (LC n-3 PUFA), such as eicosapentaenoic acid (20:5 n-3, EPA) and docosahexaenoic acid (22:6 n-3, DHA), are well-known anti-inflammatory agents (21, 22) and can serve to mitigate excessive inflammatory adipocyte–macrophage paracrine interactions *in vitro* (23, 24). Although it has been shown that LC n-3 PUFA lessen inflammatory adipokine secretion (25) and M1 macrophage polarization in obese rodent AT (26–29), the mechanisms by which this occurs are unclear. Furthermore, while LC n-3 PUFA upregulate secretion of Ad in murine (30) and human adipocytes (31), it is unclear if Ad partly mediates the anti-inflammatory effects of LC n-3 PUFA.

Thus, to assess the potential role of Ad in LC n-3 PUFA-mediated anti-inflammatory effects on ATMs, we used an established *ex vivo* AT organ culture method (32) composed of visceral AT from mice fed a control (CON) safflower oil-derived n-6 PUFA-rich diet or a fish oil (FO)-derived LC n-3 PUFA-rich diet to generate AT conditioned media (ACM). We then evaluated if CON or FO ACM affected macrophage polarization markers in an acute inflammation model in the absence or presence of Ad-neutralizing antibody. Second, we used a low-grade chronic inflammation model whereby visceral AT organ cultures from CON and FO diet fed mice were challenged with LPS *ex vivo* for 24 h prior to incubation with macrophages to mimic the chronic inflammation of metabolic endotoxemia (11, 33). Additionally, we utilized both an Ad-neutralizing antibody and the LPS-neutralizing agent, polymyxin B, to assess if Ad or LPS driven mechanisms affect LC n-3 PUFA-mediated anti-inflammatory effects in macrophages treated with ACM in the chronic inflammation model.

## Materials and Methods

### Animals and Diets

All experimental procedures were approved by the University of Guelph Animal Care Committee. Mice were housed as described (34). Eight-week-old male C57BL/6 mice were fed *ad libitum* an AIN-93G modified diet containing either 10% w/w safflower oil (CON) or an isocaloric LC n-3 PUFA-enriched diet containing 3% w/w menhaden oil + 7% w/w safflower oil (FO) for 4 weeks (*n* = 5 mice/diet), [Research Diets Inc., USA; composition previously reported (34)]. To prevent diet oxidation the common food anti-oxidant, *t*-Butylhydroquinone, was added to the diets, diets were stored at −20°C prior to use, and mouse food was changed every 2 days to limit oxidation. The FO diet contained approximately 1.7% kcal from LC n-3 PUFA, which is in line with the human dietary recommended intake of 0.5–2% kcal for total n-3 PUFA (35).

### Tissue Collection and AT Organ Culture

**Figure 1:**
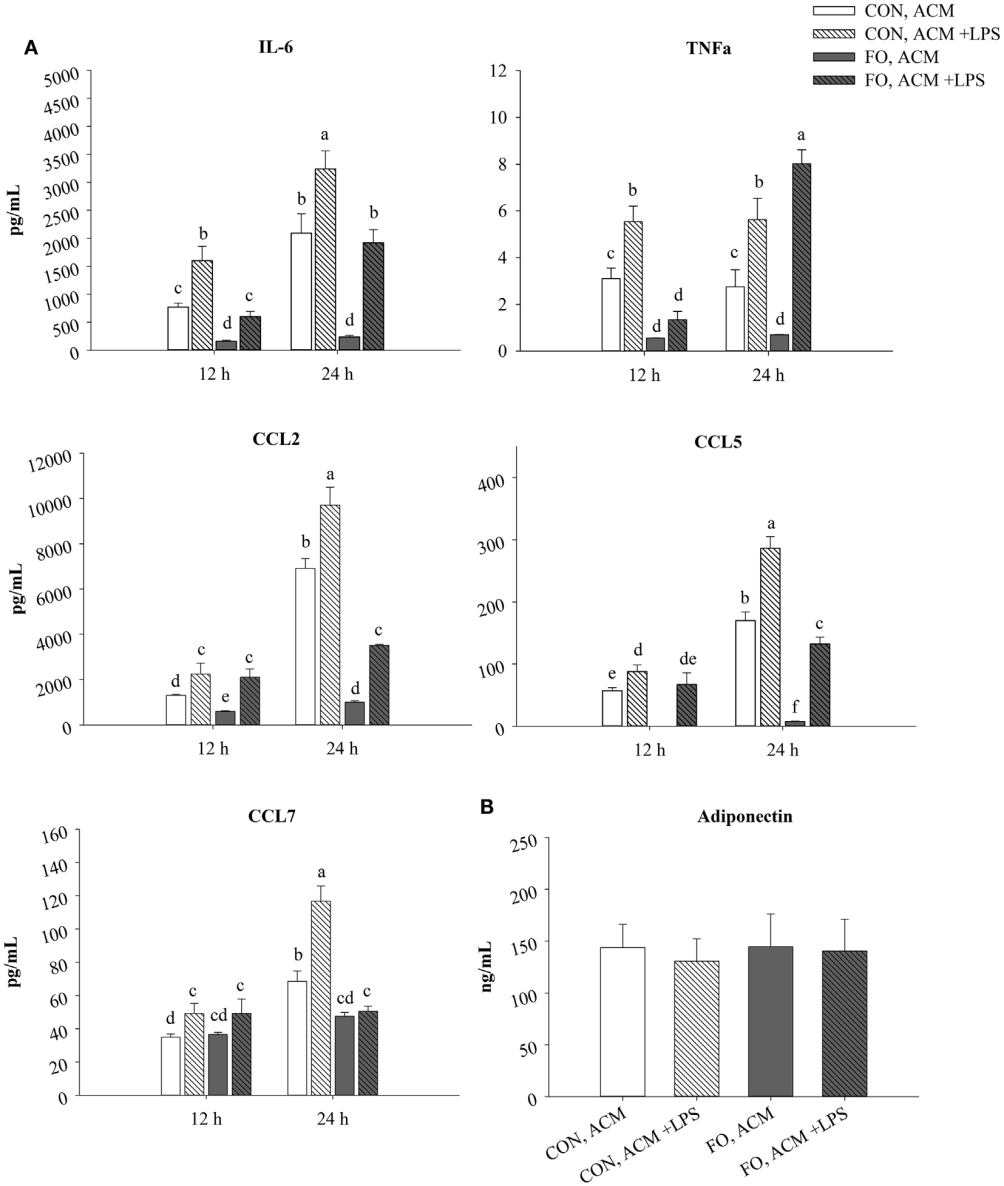
**Time course secretion of adipokines from CON and FO ACM at 12 and 24 h**. The secretion of M1-associated adipokines IL-6, TNFα, CCL2, CCL5, CCL7 **(A)** and adiponectin **(B)** from CON and FO ACM at 12 and 24 h time points ± LPS challenge. AT organ cultures from epididymal AT of CON or FO fed mice were treated with or without low-dose LPS (10 ng/mL) for 24 h. Data show the means ± SEM for CON and FO (*n* = 5/diet/condition). Bars not sharing a letter are significantly different (*p* ≤ 0.05).

Mice were terminated by CO_2_ asphyxiation followed by cervical dislocation. Epididymal fat pads were isolated and immediately placed into 50 mL sterile conical tubes containing 1× PBS (Sigma, USA). Fat pads were blotted dry, approximately cut into two equal portions, and then weighed. Tissue was divided into two per depot so that one sample could be challenged with low-dose LPS (10 ng/mL) from *E. coli* serotype 055:B5 (Sigma, USA, cell culture tested, one lot used) intended to mimic the 5–6 endotoxin units reported in metabolic endotoxemia (11, 33). Immediately after weighing, fat pads were moved to 15 mL sterile conical tubes containing 3 mL of DMEM high glucose (without sodium pyruvate, HyClone, USA) supplemented with 0.5% v/v fetal bovine serum (sterile filtered, Canadian origin, Sigma, USA); a source of anti-oxidants *in vitro* (36), and 1% v/v penicillin–streptomycin (HyClone, USA). Each tissue sample was immediately minced using sterile scissors into ≤0.5 cm pieces to avoid tissue hypoxia. Under sterile conditions, media was then topped up so that the ratio of tissue to media was at the optimal ratio of 500 mg/15 mL media as reported in the optimized method elsewhere (32). To the tissues challenged with LPS, an LPS working solution was added to the culture media such that the final concentration was 10 ng/mL. AT samples were placed into 100 mm sterile culture dishes (Sarstedt, USA) and then cultures were placed in a humidified incubator for 24 h at 37°C with 5% CO_2_. Twenty-four hours were chosen since secreted cytokines were elevated between 12 and 24 h (Figure 1). After the incubation period, the minced AT was removed using 70 μm sterile cell strainers (BD Biosciences, USA), and the ACM was aliquoted and stored at −80°C until further analysis.

### Preparation of Macrophage ACM Treatments

**Figure 2:**
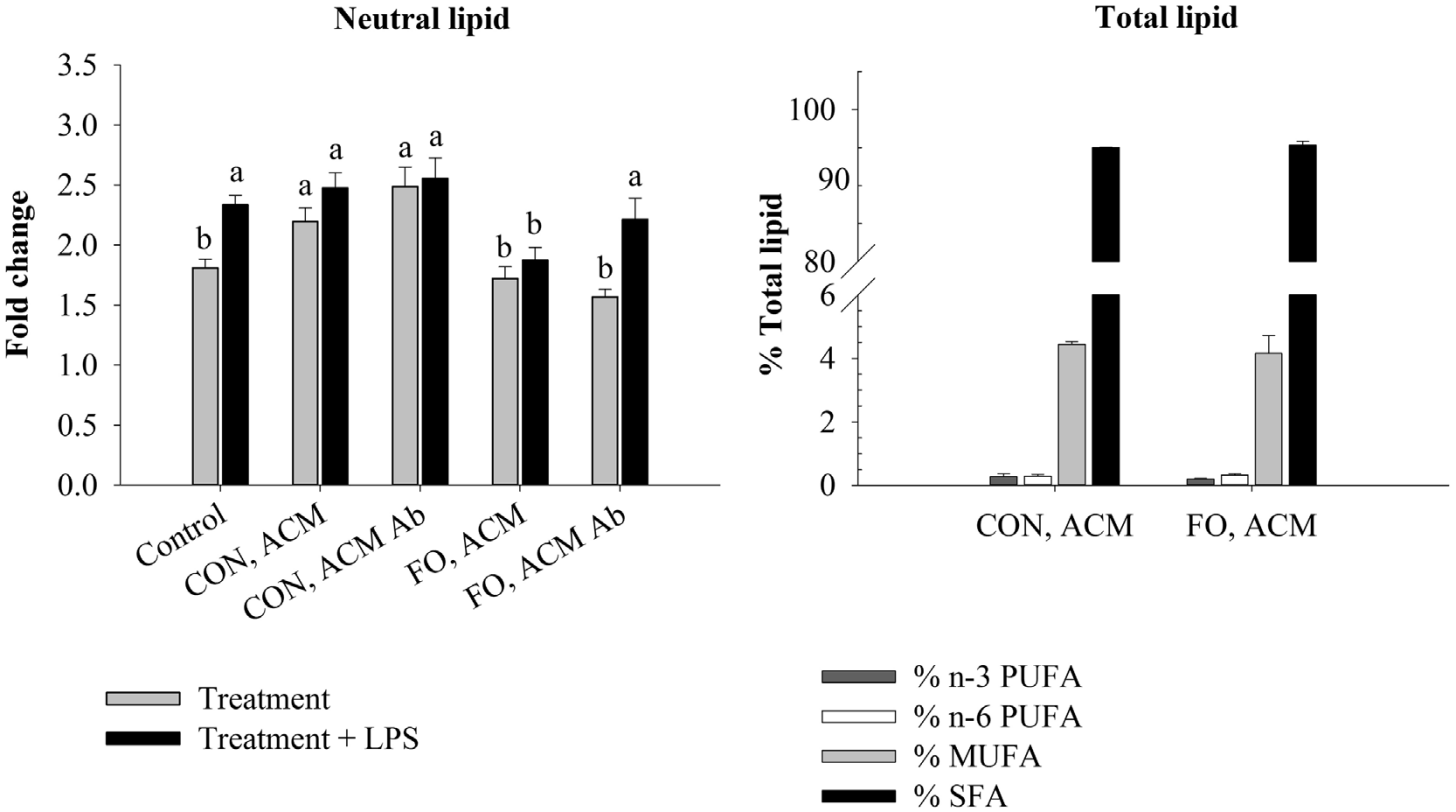
**Neutral lipid and total lipid profile in macrophages in the acute inflammation model**. Macrophages were treated with CON or FO ACM (no LPS) for 18 h, followed by the addition of low-dose LPS (10 ng/mL) for the last 6 h (24 h total). 5 μg/mL of anti-Ad or IgG were added to ACM treatments prior to culture with macrophages. Macrophages treated with media and 5 μg/mL IgG plus LPS served as the control. Data show the means ± SEM (*n* = 5/diet/condition). Bars not sharing a letter are significantly different (*p* ≤ 0.05).

**Figure 3:**
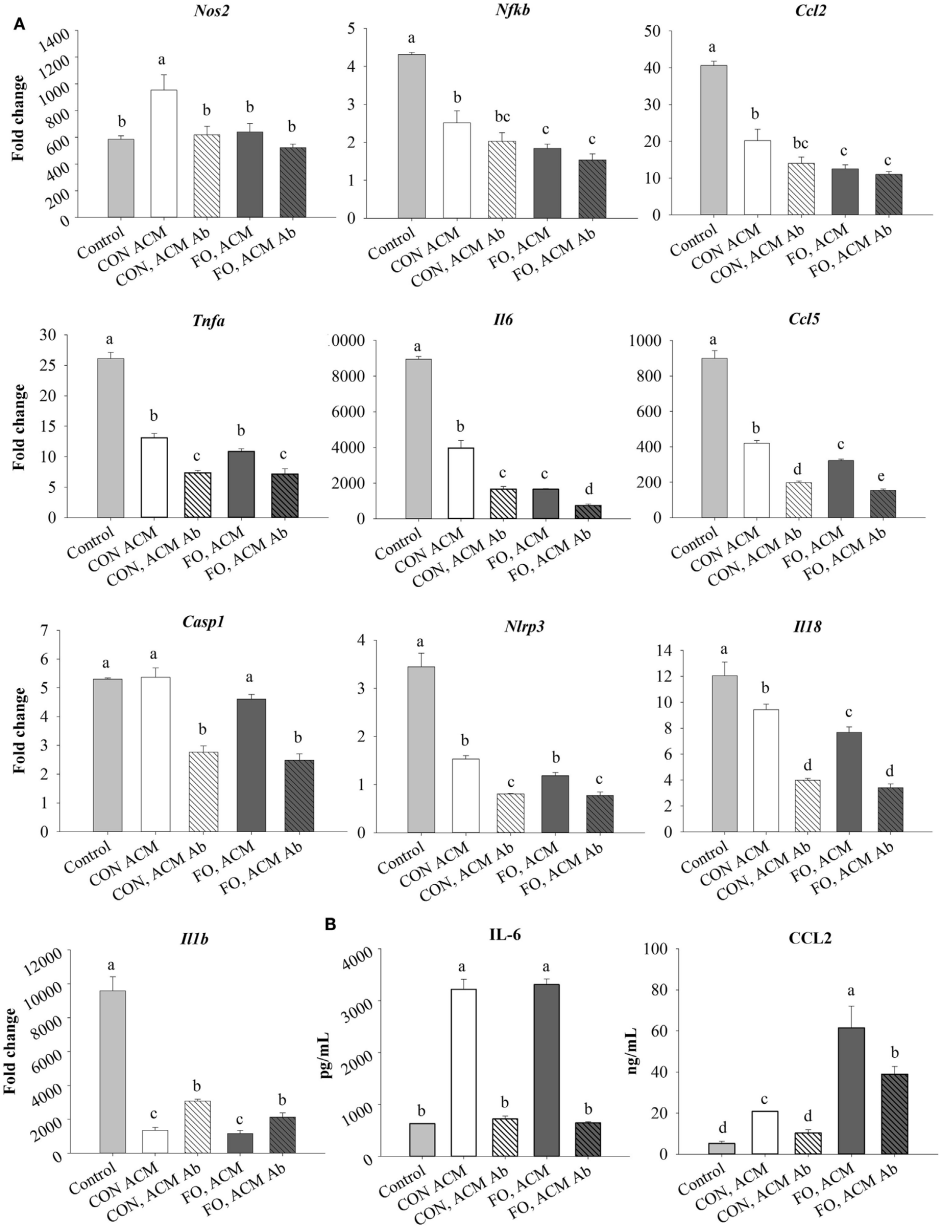
**mRNA expression of M1 markers *Nos2*, *Nf*κ*b*, *Ccl2*, *Tnf*α, *Il6*, *Ccl5*, NLRP3 inflammasome genes (*Casp1*, *Il18*, and *Il1*β) (A), and secretion of M1-associated cytokines IL-6 and CCL2 (B) from macrophages treated with ACM ± anti-Ad in the acute inflammation model**. Macrophages were treated with CON or FO ACM (no LPS) for 18 h, followed by the addition of low-dose LPS (10 ng/mL) for the last 6 h (24 h total). 5 μg/mL of anti-Ad or IgG were added to ACM treatments prior to culture with macrophages. Macrophages treated with media with 5 μg/mL IgG plus LPS served as the control. Data show the means ± SEM (*n* = 5/diet/condition). Bars not sharing a letter are significantly different (*p* ≤ 0.05).

**Figure 4:**
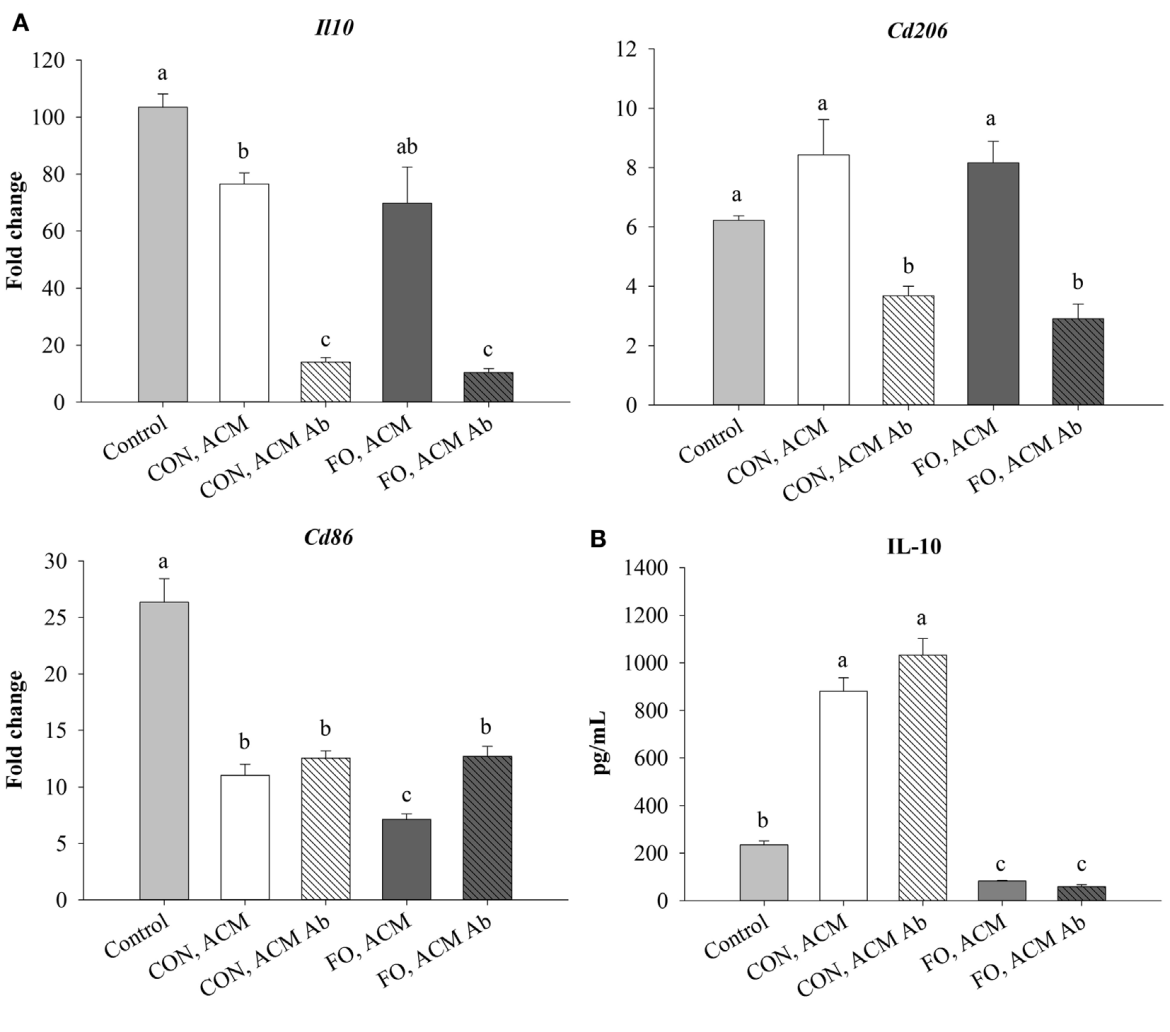
**mRNA expression of M2-associated markers *Il10*, *Cd206*, and *Cd86* (A) and secretion of IL-10 (B) from macrophages treated with ACM ± anti-Ad in the acute inflammation model**. Macrophages were treated with CON or FO ACM (no LPS) for 18 h, followed by the addition of low-dose LPS (10 ng/mL) for the last 6 h (24 h total). 5 μg/mL of anti-Ad or IgG were added to ACM treatments prior to culture with macrophages. Macrophages treated with media and 5 μg/mL IgG + LPS served as the control. Data show the means ± SEM (*n* = 5/diet/condition). Bars not sharing a letter are significantly different (*p* ≤ 0.05).

**Figure 5:**
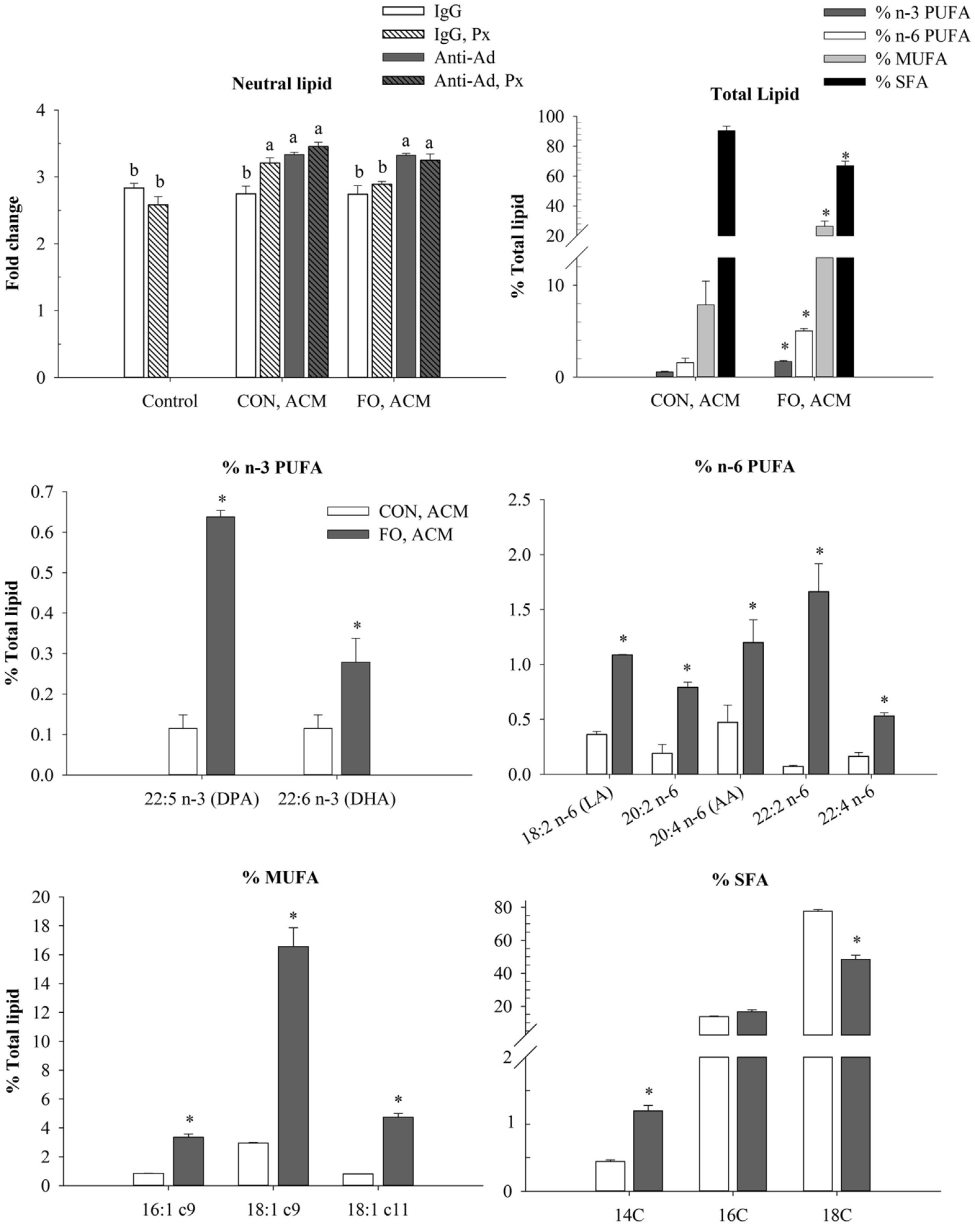
**Neutral lipid uptake and total lipid profile in macrophages treated with ACM ± anti-Ad and Px in the chronic inflammation model**. Macrophages were treated with ACM from LPS-challenged CON or FO AT organ cultures for 24 h. 5 μg/mL of anti-Ad (or IgG) ± polymyxin B (36 μM; Px) were added to ACM treatments prior to culture with macrophages. Macrophages treated with media and 5 μg/mL IgG plus 10 ng/mL LPS ± Px served as the control. Data show the means ± SEM (*n* = 5/diet/condition). Bars not sharing a letter are significantly different (*p* ≤ 0.05). An asterisk indicates that the CON group is significantly different than the respective FO group (*p* ≤ 0.05).

**Figure 6:**
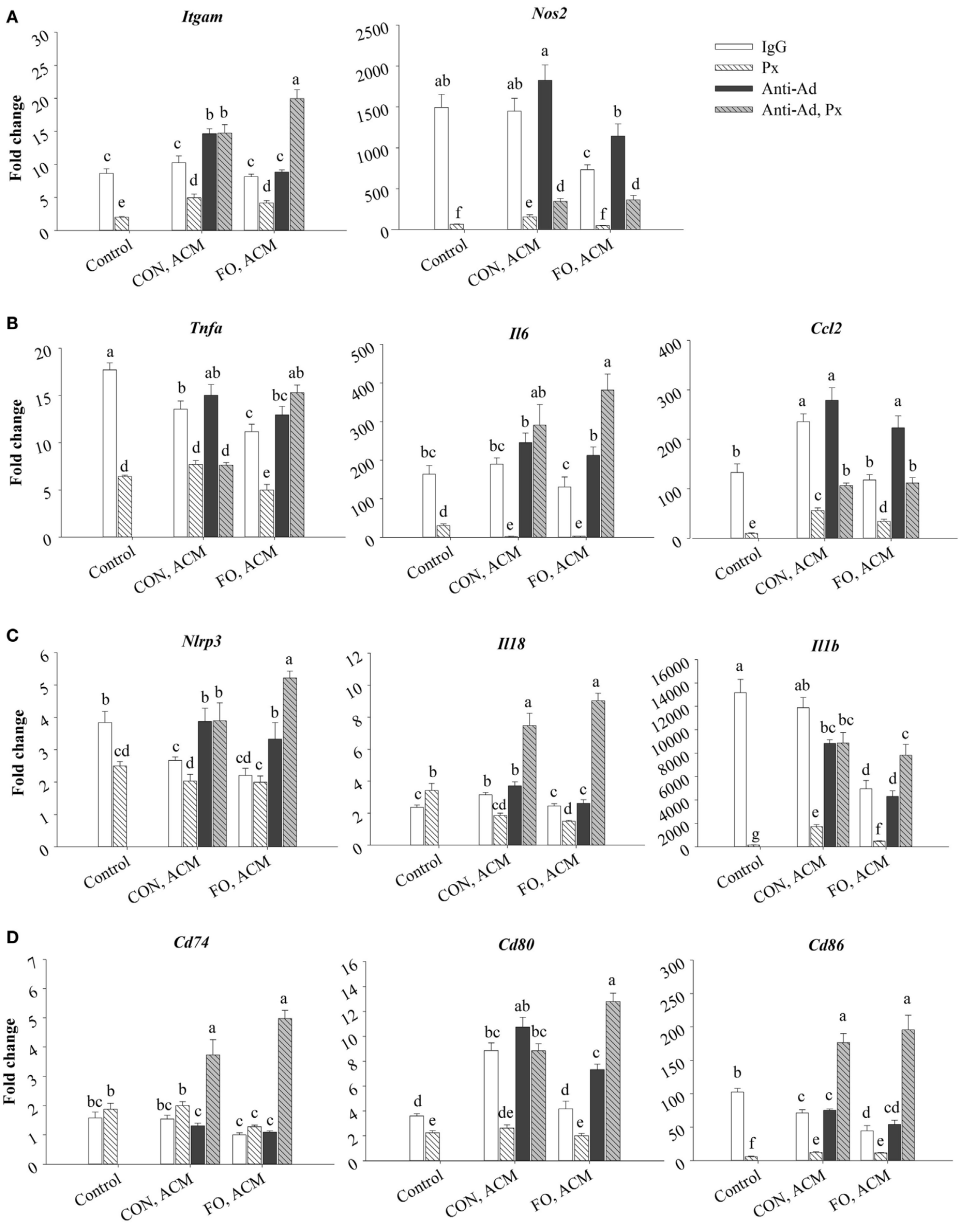
**mRNA expression of M1 markers *Itgam* and *Nos2* (A), associated cytokines *Tnf*α, *Il6*, and *Ccl2* (B), NLRP3 inflammasome genes *Nlrp3*, *Il18*, and *Il1*β (C), and key MHCII-related antigen presentation genes *Cd74*, *Cd80*, and *Cd86* (D) from macrophages treated with ACM ± anti-Ad and Px in the chronic inflammation model**. Macrophages were treated with ACM from LPS-challenged (10 ng/mL) CON or FO AT organ cultures for 24 h. 5 μg/mL of anti-Ad (or IgG) ± polymyxin B (36 μM; Px) were added to ACM treatments prior to culture with macrophages. Macrophages treated with media and 5 μg/mL IgG and 10 ng/mL LPS ± Px served as the control. Data show the means ± SEM (*n* = 5/diet/condition). Bars not sharing a letter are significantly different (*p* ≤ 0.05).

**Figure 7:**
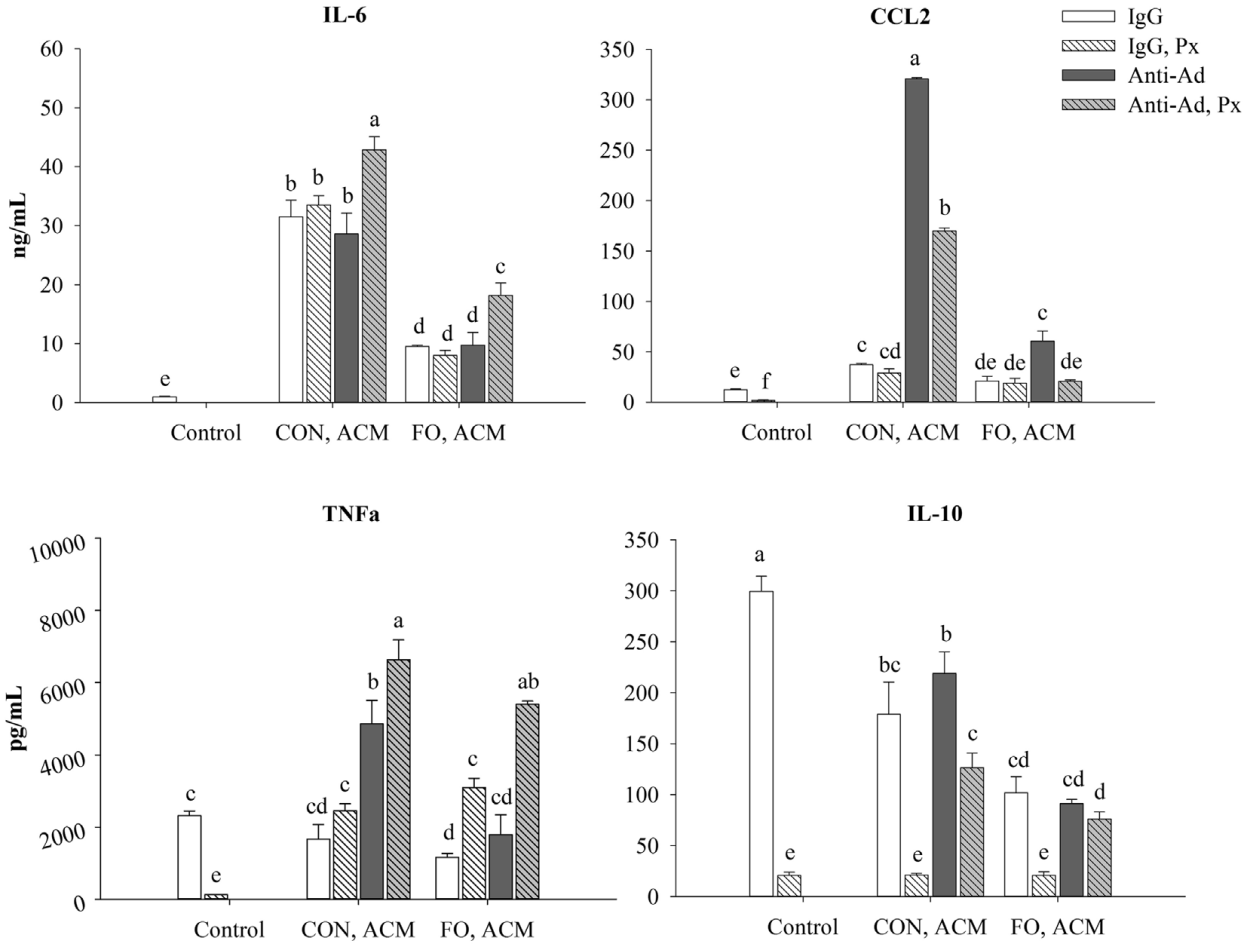
**Secretion of M1-associated cytokines (IL-6, CCL2, and TNFα) and anti-inflammatory cytokine, IL-10 from macrophages treated with ACM ± anti-Ad and Px in the chronic inflammation model**. Macrophages were treated with ACM from LPS-challenged (10 ng/mL) CON or FO AT organ cultures for 24 h. 5 μg/mL of anti-Ad (or IgG) ± polymyxin B (36 μM; Px) were added to ACM treatments prior to culture with macrophages. Macrophages treated with media and 5 μg/mL IgG and 10 ng/mL LPS ± Px served as the control. Data show the means ± SEM (*n* = 5/diet/condition). Bars not sharing a letter are significantly different (*p* ≤ 0.05).

The ACM was thawed once and then sterile filtered (0.22 μm, Sarstedt, USA) prior to use. Before incubation with macrophages, 5 μg/mL of Ad-neutralizing antibody (AF1119, anti-mAcrp30, R&D Systems, USA) or isotype control anti-goat IgG (5 μg/mL, R&D Systems, USA) was added to ACM. The samples were then incubated at 4°C as previously reported (37). This antibody was chosen since it has been reported to suppress Ad-mediated signaling in adipocyte-conditioned media (37, 38), and we confirmed the dose of antibody used suppressed Ad-mediated signaling in pilot experiments with adipocyte-conditioned media (data not shown). In the acute inflammation model (Figures 2–4), macrophages were pre-treated with ACM from CON or FO fed mice (no LPS) for 18 h, followed by the addition of LPS (10 ng/mL) to the ACM for the final 6 h (total 24 h). This time course was chosen since both globular (18) and full-length Ad (15) have previously been shown to promote a response similar to endotoxin tolerance using a comparable time course. In the chronic inflammation model (Figures 5–7), visceral AT organ cultures from CON and FO diet fed mice were challenged with LPS *ex vivo* for 24 h prior to incubation with macrophages for an additional 24 h. Ad-neutralizing antibody was added to ACM as described above. Additionally, the LPS-neutralizing agent, polymyxin B (36 μM, 0.22 μm sterile filtered, cell-culture tested, Sigma, USA), was added to some ACM treatments with gentle vortexing every 10 min for a total of 30 min prior to administering ACM to macrophages at 37°C. For all experiments, macrophages treated with media containing 5 μg/mL IgG plus 10 ng/mL LPS served as the control.

### Culture of RAW 264.7 Macrophages

RAW 264.7 murine macrophages (ATCC, USA) were grown and passaged according to the manufacturer’s instructions. Macrophages were maintained in DMEM high glucose (without sodium pyruvate, HyClone, USA), plus 10% v/v fetal bovine serum (low-endotoxin, Canadian origin, Sigma, USA), and 1% v/v penicillin streptomycin (HyClone, USA). Four hours prior to experiments, macrophages were split using the manufacturer’s protocol, counted using trypan exclusion, spun down at 335 × *g* at ambient temperature for 5 min, and then re-suspended in DMEM high glucose plus 0.5% v/v FBS and 1% v/v penicillin streptomycin. Next, the cell density was adjusted to 1.0 × 10^6^ cells/mL and macrophages were seeded into 96-well (for oil red O procedure) or 24-well plates (for RNA/protein analysis) (Corning, USA). Without disturbing the adherent macrophages, media was replaced with the aforementioned control, CON or FO ACM treatments.

### Gene Expression

After 24 h, cells were washed with 1× PBS, lysed using a RNA/Protein Isolation Kit (Norgen Biotek, Canada), and processed according to the manufacturer’s instructions. cDNA was made from 1 μg of extracted RNA using a high capacity cDNA reverse transcription kit as per the manufacturer’s instructions (Applied Biosystems, USA). Real-time PCR analysis was performed using a 7900HT Fast Real-Time PCR system (Applied Biosystems, USA) using the default protocol described elsewhere (23). Primers were designed using the Universal Probe Library Assay Design Center (Roche Applied Sciences, Germany, Table 1) and validated primer efficiencies were between 90 and 105%. All results were normalized to *18S* mRNA expression, and the relative differences in gene expression between treatment groups and the IgG control (no LPS) were determined using the ΔΔCt method.

**Table 1:** **Primer sequences**.

Gene name	Forward primer (5′–3′)	Reverse primer (3′–5′)
*18S*	ACGGAAGGGCACCACCAGGA	CACCACCACCCACGGAATCG
*Casp1*	CCCACTGCTGATAGGGTGAC	GCATAGGTACATAAGAATGAACTGGA
*Ccl2*	GCCTGCTGTTCACAGTTGC	CAGGTGAGTGGGGCGTTA
*Ccl5*	TCCAATCTTGCAGTCGTGTTTG	TCTGGGTTGGCACACACTTG
*Cd206*	CCACAGCATTGAGGAGTTTG	ACAGCTCATCATTTGGCTCA
*Cd74*	CGCCCTAGAGAGCCAGAAA	TTGCTGGTACAGGAAGTAAGCA
*Cd80*	TCGTCTTTCACAAGTGTCTTCAG	TTGCCAGTAGATTCGGTCTTC
*Cd86*	GAAGCCGAATCAGCCTAGC	CAGCGTTACTATCCCGCTCT
*Il6*	AACGATGATGCACTTGCAGA	GAGCATTGGAAATTGGGGTA
*Il10*	GGTTGCCAAGCCTTATCGGA	ACCTGCTCCACTGCCTTGCT
*Il1*β	AGTTGACGGACCCCAAAAG	AGCTGGATGCTCTCATCAGG
*Il18*	CAAACCTTCCAAATCACTTCCT	TCCTTGAAGTTGACGCAAGA
*Itgam*	AGCCCCACACTAGCATCAA	TCCATGTCCACAGAGCAAAG
*Nf*κ*b*	GAGACCGGCAACTCAAGAC	CTCAGGTCCATCTCCTTGGGT
*Nlrp3*	CCCTTGGAGACACAGGACTC	GAGGCTGCAGTTGTCTAATTCC
*Nos2*	TCCTGTTGTTTCTATTTCCTTTGTT	CATCAACCAGTATTATGGCTCCT
*Tnf*α	CATCTTCTCAAAATTCGAGTGACAA	TGGGAGTAGACAAGGTACAACCC

### Secreted Adipokine Analyses

Supernatant was collected after 12 and 24 h from ACM, and after 24 h following ACM culture with macrophages for analysis of secreted cytokine concentrations. CCL2 (MCP-1), CCL5 (RANTES), CCL7 (MCP-3), IL-4, IL-6, IL-10, IL-18, IL-1β, and TNFα were multiplexed using the ProcartaPlex Mouse Basic kit (eBioscience, USA) using undiluted supernatant and analyzed using the Bio-Plex 200 System (Bio-Rad, USA). To calculate the secretion of adipokines solely from macrophages after 24 h treatment with ACM, the concentration of cytokine measured in ACM prior to culture with macrophages was subtracted from the concentration of cytokine measured after the incubation. Importantly, cell protein and secreted levels of IL-1β and IL-18 were below the minimum detection threshold for all treatments. Additionally, secreted full-length Ad was diluted 100-fold and measured by ELISA (Quantikine Mouse Ad/Acrp 30 ELISA, R&D Systems, USA) according to the manufacturer’s instructions.

### Macrophage Fatty Acid Composition

Following 24 h, macrophages were scraped in 1× PBS and then centrifuged at 335 × *g* at ambient temperature for 10 min. The PBS was aspirated and the pellet was frozen at −80°C until further processing. Total lipids were extracted from cell lysates of macrophages and fatty acid methyl esters were prepared for analysis by gas liquid chromatography as described previously (39, 40). Fatty acids were identified by comparing the retention times of samples with those of a known standard (GLC463; Nu-Chek Prep, USA). Fatty acid composition values are expressed as a percentage of total fatty acids. Within each ACM treatment, the lipid profile between macrophages treated with Ad-neutralizing antibody to those treated with IgG did not differ; therefore, only ACM treatments (IgG) are shown (Figures 2 and 5).

### Macrophage Oil Red O Procedure

Following the 24 h ACM treatment, neutral lipid deposition was assessed in macrophages with minor modification from the original procedure (41). Briefly, an oil red O working solution was made 6 h in advance by mixing 6 parts oil red O saturate (0.5 g in 100 mL isopropyl alcohol) in 4 parts ddH_2_O, and then filtered twice immediately before use. Next, media was aspirated and cells were then rinsed with 1× PBS. Cultures were fixed with 4% paraformaldehyde solution followed by light shaking at ambient temperature for 10 min. Cultures were then sequentially rinsed with 1× PBS and 70% ethanol before staining with oil red O working solution for 30 min (at ambient temp with light shaking). Following this, cultures were then rinsed again with 1× PBS, then 70% ethanol solution. The solutions were then aspirated and the plate was inverted to dry in a laminar flow hood for 30 min. Next, 200 μL of isopropyl alcohol was added to each well. The plate was sealed, shook lightly for 3 min, and then read immediately with a spectrophotometer at 520 nm. Wells without cells that received the aforementioned staining procedure served as the plate blank. Data are reported as the fold change in absorbance (per treatment) relative to the average absorbance for cells cultured in media without LPS or other additives (*n* = 4).

### Statistical Analysis

All data are expressed as mean ± SEM. The predetermined upper limit of probability for statistical significance was *p* ≤ 0.05 and analyses were conducted using SigmaPlot version 12.5 (USA). Data that were not normally distributed were transformed prior to statistical analysis, and normal distribution and equal variance were confirmed by Shapiro–Wilk test and Levene’s test, respectively. Data were analyzed using a two-way ANOVA followed, if justified, by testing using Fisher’s Least Squared Difference *post hoc* test. Mouse parameters comparing CON and FO diets (Table 2) and macrophage lipid content between CON and FO ACM (Figures 2 and 5) were analyzed using unpaired *t*-tests.

**Table 2:** **Mouse physiological parameters^a^**.

Parameter	CON	FO
Initial body weight (g)	21.0 ± 0.63	21.8 ± 0.52
Terminal body weight (g)	25.0 ± 2.2	27.5 ± 1.2
Food intake (g/day)	2.24 ± 0.04	2.22 ± 0.03
Epididymal fat pad weight (g)	1.36 ± 0.27	1.45 ± 0.15

*^a^Values are means ± SEM (n = 5/dietary group)*.

## Results

### Mouse Parameters

Mouse initial and terminal body weights, dietary food intake, and epididymal fat pad weights did not differ between mice fed CON or FO diets (*p* > 0.05, Table 2). Previous studies using these diets showed that LC n-3 PUFA are significantly enriched in liver and red blood cells (42).

### ACM Secretory Profile After 24 h With or Without LPS

In CON ACM, IL-6, TNFα, CCL2, CCL5, and CCL7 secretion increased in the presence of LPS at both 12 and 24 h (Figure 1). Similarly, in FO ACM, IL-6, CCL2, and CCL5 secretion increased when LPS was added at both 12 and 24 h; however, TNFα secretion only increased at 24 h (*p* ≤ 0.05, Figure 1). Moreover, in FO ACM, secretion of CCL7 did not change over time or with LPS (*p* > 0.05, Figure 1). Interestingly, in FO ACM without LPS, the secretion of IL-6, TNFα, CCL2, and CCL5 were decreased relative to CON ACM at both 12 and 24 h; however, CCL7 was only decreased at 24 h (*p* ≤ 0.05, Figure 1). Similarly, relative to CON ACM with LPS, in FO ACM with LPS the secretion of IL-6 was decreased at both 12 and 24 h, although the secretion of other adipokines was only decreased at one time point (CCL2, CCL5, and CCL7 at 24 h; TNFα at 12 h), (*p* ≤ 0.05, Figure 1). Furthermore, the secretion of full-length Ad in ACM did not vary with diet or LPS (*p* > 0.05, Figure 1). Finally, IL-1β, IL-18, IL-4, and IL-10 concentrations were below the range of detection in all ACM treatments.

### Lipid Profile in Macrophages Treated With ACM in the Acute Inflammation Model

To examine if the Ad within ACM from CON or FO fed mice could promote a response similar to endotoxin tolerance, macrophages were pre-treated with ACM or media for 18 h with Ad-neutralizing antibody (anti-Ad) or control IgG, then LPS was added for 6 h for a total of 24 h. First, we measured total lipid uptake using oil red O staining to measure the relative abundance of neutral lipids, and then quantified the percent of total lipids (n-3 and n-6 PUFA, MUFA, and SFA) within the macrophages treated for 24 h with CON or FO ACM (Figure 2). Here, without LPS, macrophages treated with CON ACM had increased neutral lipid relative to control (*p* ≤ 0.05); however, no such increase occurred in macrophages treated with FO ACM (*p* > 0.05, Figure 2). Second, adding LPS resulted in an increase in neutral lipid in control macrophages (*p* ≤ 0.05, Figure 2). However, levels of neutral lipid did not change when LPS was added in macrophages treated with CON or FO ACM (*p* > 0.05, Figure 2). Interestingly, when LPS was added to ACM with anti-Ad, levels of neutral lipid increased in macrophages treated with FO ACM (*p* ≤ 0.05), but not CON ACM (*p* > 0.05, Figure 2). Finally, the percent of n-3 PUFA (0.24 ± 0.04%), n-6 PUFA (0.33 ± 0.03%), MUFA (4.30 ± 0.24%) and SFA (95.2 ± 0.22%) did not differ in macrophages treated with CON or FO ACM in this model (*p* > 0.05, Figure 2).

### mRNA Expression of M1 Markers and Associated Secreted Cytokines in Macrophages Treated With ACM in the Acute Inflammation Model

First, treating macrophages with low-dose LPS for 6 h (control) induced the expression of M1 markers and NLRP3 inflammasome-related genes shown in Figure 3 relative to macrophages treated with just IgG (data not shown, *p* ≤ 0.05). Second, treating macrophages with either CON or FO ACM induced a response similar to endotoxin tolerance at the mRNA level as evidenced by decreased expression of M1 markers, *Nf*κ*b*, *Ccl2*, *Tnf*α, *Il6*, and *Ccl5*, as well as NLRP3 inflammasome-associated genes *Nlrp3*, *Il18*, and *Il1*β (Figure 3A, *p* ≤ 0.05). By contrast, macrophages treated with CON ACM had increased mRNA expression of *Nos2* relative to control (Figure 3A, *p* ≤ 0.05), whereas mRNA expression of *Casp1* in macrophages treated with CON or FO ACM did not differ from control (Figure 3A, *p* > 0.05). Second, when anti-Ad was added, within each respective ACM group, the mRNA expression of M1 markers, *Nos2* (CON ACM only), *Tnf*α, *Il6*, and *Ccl5*, as well as NLRP3 inflammasome-related genes, *Casp1*, *Nlrp3*, and *Il18* (Figure 3A), decreased (*p* ≤ 0.05). By contrast, relative to each respective ACM treatment group, mRNA expression of *Il1*β increased with anti-Ad (*p* ≤ 0.05, Figure 3A). Third, mRNA expression of M1 genes, *Nos2* (FO ACM only), *Nf*κ*b*, and *Ccl2* decreased in macrophages treated with ACM regardless of adding anti-Ad (Figure 3A). Additionally, relative to CON ACM, macrophages treated with FO ACM had decreased mRNA expression of *Nos2*, *Nf*κ*b*, *Ccl2*, *Il6*, *Ccl2*, and *Il18* (*p* ≤ 0.05), although this effect was lost for *Nos2*, *Nf*κ*b*, *Ccl2*, and *Il18* with anti-Ad (*p* > 0.05, Figure 3A). Fourth, secretion of IL-6 and CCL2 increased in both CON and FO ACM-treated macrophages relative to control; however, with anti-Ad, secretion of IL-6 and CCL2 (CON ACM only) returned to control levels (*p* > 0.05, Figure 3B). Interestingly, in FO ACM-treated macrophages, secretion of CCL2 was elevated above all treatment groups, although this was partly subdued with anti-Ad (*p* ≤ 0.05, Figure 3B).

### mRNA Expression of M2 Markers and Secreted IL-10 in Macrophages Treated With ACM in the Acute Inflammation Model

Treating macrophages with low-dose LPS for 6 h (control) induced mRNA expression of M2 markers, *Il10* and *Cd206*, and the antigen presentation co-stimulatory molecule, *Cd86*, relative to macrophages treated with just IgG (data not shown, *p* ≤ 0.05). Macrophages treated with CON ACM had decreased mRNA expression of *Il10* relative to control, while macrophages treated with both CON and FO ACM had decreased *Cd86* relative to control (*p* ≤ 0.05, Figure 4A). However, when anti-Ad was added to CON and FO ACM, mRNA expression of both *Il10* and *Cd206* were decreased (*p* ≤ 0.05, Figure 4A). Third, mRNA expression of *Cd86* in macrophages treated with FO ACM was decreased relative to CON ACM, though this effect was lost with anti-Ad (*p* ≤ 0.05, Figure 4A). Finally, IL-10 secretion was increased (*p* ≤ 0.05) in macrophages treated with CON ACM relative to control regardless of adding anti-Ad (Figure 4B). By contrast, IL-10 secretion decreased in macrophages treated with FO ACM relative to control and CON ACM (*p* ≤ 0.05), and this occurred regardless of adding anti-Ad (Figure 4B).

### Lipid Profile in Macrophages Treated With ACM in the Chronic Inflammation Model

To examine if the Ad within ACM from CON or FO fed mice could affect macrophage phenotype in a chronic inflammation model, macrophages were treated with ACM collected from LPS-challenged visceral AT organ cultures or control media for 24 h with anti-Ad or control IgG. Additionally, within ACM samples, LPS was neutralized by polymyxin B (Px) to test if Ad worked synergistically with LPS. First, in macrophages treated with CON or FO ACM, we measured the relative abundance of neutral lipids after 24 h using oil red O staining and then quantified the percent of total n-3 and n-6 PUFA, MUFA and SFA relative to total fatty acid content (only showing those above 0.2% trace amounts of total lipid) (Figure 5). Interestingly, macrophages treated with CON ACM had similar neutral lipid relative to control; however, neutral lipid increased when Px, anti-Ad, or anti-Ad plus Px were added to CON ACM (*p* ≤ 0.05, Figure 5). By contrast, macrophages treated with FO ACM only had elevated levels of neutral lipid compared to control when anti-Ad or anti-Ad plus Px were added to FO ACM (*p* ≤ 0.05, Figure 5). Second, compared to macrophages treated with CON ACM, macrophages treated with FO ACM had increased total n-3 PUFA, n-6 PUFA and MUFA, while total SFA decreased (*p* ≤ 0.05, Figure 5). More specifically, macrophages treated with FO ACM had increased n-3 PUFA, including docosapentaenoic acid (DPA, 22:5 n-3) and docosahexaenoic acid (DHA, 22:6 n-3); increased n-6 PUFA, including linoleic acid (LA, 18:2 n-6) and arachidonic acid (AA, 20:4 n-6); and increased MUFA, including palmitoleic (16:1 c9), oleic (18:1 c9), and vaccenic (18:1 c11) acid (*p* ≤ 0.05, Figure 5). With regards to SFAs, macrophages treated with FO ACM also had increased myristic acid (14:0), while stearic acid (18:0) was decreased (*p* ≤ 0.05, Figure 5).

### mRNA Expression of M1 Markers in Macrophages Treated With ACM in the Chronic Inflammation Model

Treating macrophages with low-dose LPS for 24 h (control) induced the expression of M1 markers relative to macrophages treated with just IgG (data not shown, *p* ≤ 0.05), and adding Px to media with LPS suppressed this expression (*p* ≤ 0.05, Figure 6A). First, *Itgam* (CD11b) and *Nos2* were similarly expressed in control macrophages and macrophages treated with CON ACM; however, mRNA expression of *Nos2* decreased in FO ACM-treated macrophages (*p* ≤ 0.05, Figure 6A). Interestingly, with anti-Ad added, mRNA expression of *Itgam* in CON ACM increased above control levels (*p* ≤ 0.05, Figure 6A). Similarly, with anti-Ad in FO ACM, expression of *Nos2* increased to be similar to the control group (*p* > 0.05, Figure 6A). Second, adding Px to ACM decreased mRNA expression of *Itgam* and *Nos2* in each respective ACM group; however, when both anti-Ad and Px were added to ACM, mRNA expression of *Itgam* and *Nos2* increased compared to each respective ACM group with Px (*p* ≤ 0.05, Figure 6A). Intriguingly, in the FO ACM treatment with anti-Ad and Px, mRNA expression of *Itgam* increased above all other treatment groups (*p* ≤ 0.05, Figure 6A). Finally, with regard to differences between CON and FO ACM groups, mRNA expression of *Nos2* was decreased in the FO ACM group even with Px or anti-Ad (*p* ≤ 0.05, Figure 6A).

Moreover, macrophages treated with CON ACM had decreased mRNA expression of *Tnf*α relative to control, although expression of *Il6* was similar to control, and expression of *Ccl2* was greater than control (*p* ≤ 0.05, Figure 6B). By contrast, macrophages treated with FO ACM had decreased mRNA expression of *Tnf*α and *Il6* relative to control (*p* ≤ 0.05), and expression of *Ccl2* was similar between these two groups (*p* > 0.05, Figure 6B). Second, when anti-Ad was added to each respective ACM group, increased mRNA expression of *Il6* and *Ccl2* was observed in the FO ACM group only (*p* ≤ 0.05, Figure 6B). Third, adding Px to ACM decreased the mRNA expression of *Tnf*α, *Il6*, and *Ccl2* in each respective ACM group; however, when both anti-Ad and Px were added to ACM, mRNA expression of these cytokines significantly increased in each respective ACM group with Px (with the exception of *Tnf*α expression in the CON ACM group), (*p* ≤ 0.05, Figure 6B). Finally, with regards to differences between CON and FO ACM groups, mRNA expression of *Tnf*α and *Ccl2* decreased in the FO ACM group, even with Px added (*p* ≤ 0.05); however, this relationship was lost with the addition of anti-Ad and anti-Ad plus Px to FO ACM (*p* > 0.05, Figure 6B).

### mRNA Expression of NLRP3 Inflammasome Genes in Macrophages Treated With ACM in the Chronic Inflammation Model

Treating macrophages with low-dose LPS for 24 h (control) induced the expression of NLRP3 inflammasome genes relative to macrophages treated with just IgG (data not shown, *p* ≤ 0.05), and adding Px to media with LPS (with the exception of *Il18*) suppressed this expression (*p* ≤ 0.05, Figure 6C). First, macrophages treated with CON ACM had increased mRNA expression of *Il18* relative to the control (*p* ≤ 0.05), whereas expression of *Nlrp3* was less than the control, and expression of *Il1*β was similar to control (*p* > 0.05, Figure 6C). By contrast, macrophages treated with FO ACM had decreased mRNA expression of *Nlrp3* and *Il1*β relative to the control (*p* ≤ 0.05), and expression of *Il18* did not differ from control (*p* > 0.05, Figure 6C). In each respective ACM group, adding anti-Ad resulted in increased mRNA expression of *Nlrp3* in both CON and FO ACM groups (*p* ≤ 0.05, Figure 6C). Adding Px in each respective CON or FO ACM group decreased mRNA expression of *Il18* and *Il1*β, although *Nlrp3* expression only decreased in the CON ACM group (*p* ≤ 0.05, Figure 6C). Interestingly, adding anti-Ad and Px to each respective CON or FO ACM group compared to ACM with just Px increased mRNA expression of *Nlrp3*, *Il18*, and *Il1*β (*p* ≤ 0.05, Figure 6C). Finally, with regards to differences between CON and FO ACM groups, mRNA expression of *Il18* and *Il1*β decreased with FO ACM, even with anti-Ad was added (*p* ≤ 0.05); however, this relationship was lost when anti-Ad plus Px were added to FO ACM (*p* > 0.05, Figure 6C).

### mRNA Expression of MHCII-Related Antigen Presentation Genes in Macrophages Treated With ACM in the Chronic Inflammation Model

Treating macrophages with low-dose LPS for 24 h (control) induced the expression of MHCII-related antigen presentation genes relative to macrophages treated with just IgG (data not shown, *p* ≤ 0.05), and adding Px to media with LPS (with the exception of *Cd74*) suppressed this expression (*p* ≤ 0.05, Figure 6D). First, macrophages treated with CON ACM had increased mRNA expression of *Cd80* relative to the LPS control (*p* ≤ 0.05), whereas expression of *Cd74* was similar to control (*p* > 0.05), and expression of *Cd86* was less than control (*p* ≤ 0.05, Figure 6D). Moreover, macrophages treated with FO ACM had similar levels of *Cd74* and *Cd80* expression as the control (*p* > 0.05), although expression of *Cd86* was less than the control (*p* ≤ 0.05, Figure 6D). Second, when anti-Ad was added, expression of MHCII-related genes only differed within each respective ACM group in one scenario; mRNA expression of *Cd80* increased in the FO ACM group (*p* ≤ 0.05, Figure 6D). Third, adding Px to each respective ACM group decreased mRNA expression of *Cd80* and *Cd86* in both CON and FO ACM (*p* ≤ 0.05). Interestingly, when anti-Ad plus Px were added to each respective ACM group, mRNA expression of *Cd74*, *Cd80*, and *Cd86* increased compared to ACM with Px or anti-Ad alone (with the exception of *Cd80* expression in CON ACM with anti-Ad relative to CON ACM with anti-Ad and Px) (*p* ≤ 0.05, Figure 6D). Finally, with regards to differences between CON and FO ACM groups, FO ACM groups had decreased mRNA expression of *Cd74* (between Px groups only), *Cd80* (between IgG and anti-Ad groups only), and *Cd86* (between IgG groups only), (*p* ≤ 0.05, Figure 6D).

### Secretion of Cytokines in Macrophages Treated With ACM in the Chronic Inflammation Model

First, secretion of all of the aforementioned cytokines increased in LPS-treated macrophages after 24 h compared to the IgG control (*p* ≤ 0.05, data not shown). Second, adding Px to the LPS control decreased secretion of all of the aforementioned cytokines (*p* ≤ 0.05, Figure 7). Third, relative to control macrophages, those treated with CON ACM had increased secretion of IL-6 and CCL2 (*p* ≤ 0.05), whereas TNFα was similar (*p* > 0.05), and secretion of IL-10 was less than the control (*p* ≤ 0.05, Figure 7). Moreover, compared to control, in FO ACM, secretion of CCL2 was similar, IL-6 was greater, and TNFα and IL-10 were decreased (*p* ≤ 0.05, Figure 7). Fourth, when anti-Ad was added to each respective ACM group, the secretion of CCL2 increased in both CON and FO ACM, whereas secretion of TNFα increased in the CON ACM group only (*p* ≤ 0.05, Figure 7). Fifth, when Px was added to each respective ACM group, secretion of TNFα increased in the FO ACM group, whereas IL-10 secretion decreased in both the CON and FO ACM group (*p* ≤ 0.05), and secretion of IL-6 and CCL2 did not change (*p* > 0.05, Figure 7). Sixth, with regards to differences between CON and FO ACM groups, FO ACM groups had decreased secretion of IL-6 and CCL2 even when Px (with the exception of CCL2), anti-Ad, or anti-Ad plus Px were added to ACM (*p* ≤ 0.05, Figure 7). Interestingly, when anti-Ad was added to ACM there was a decrease in secretion of TNFα and IL-10 in the FO ACM group compared to the CON ACM group (*p* ≤ 0.05); however, this effect was lost for TNFα when anti-Ad plus Px were added to FO ACM (*p* > 0.05, Figure 7).

## Discussion

Although it was known that LC n-3 PUFA modulate adipocyte–macrophage paracrine interactions (23, 24), as well as the AT secretory profile in obese mice (25), the effect of LC n-3 PUFA on the intact AT secretory profile and how this subsequently affects macrophage polarization status was not known. Thus, we generated visceral ACM from mice fed a CON (n-6 PUFA-rich) or FO (LC n-3 PUFA-rich) diet and then examined its effects on macrophage phenotype under different stimulation conditions designed to mimic aspects of the AT microenvironment. Specifically, we utilized two models: an acute inflammation model to assess if pre-treating macrophages with ACM could promote a response similar to endotoxin tolerance in macrophages when stimulated with low-dose LPS and a low-grade chronic inflammation model wherein AT organ cultures were challenged with LPS to make ACM prior to incubation with macrophages to mimic the inflammatory state that occurs in parallel with metabolic endotoxemia (11, 33). Here, we report for the first time that in the acute inflammation model, macrophages treated with FO ACM had decreased lipid uptake (Figure 2), and mRNA expression of M1-associated markers (*Nos2*, *Nf*κ*b*, *Il6*, *Il18*, *Ccl2*, and *Ccl5*, Figure 3) compared with CON ACM; however, these effects were largely attenuated when Ad was neutralized, indicating that Ad is an important factor in LC n-3 PUFA-mediated effects. Furthermore, intact AT from mice fed CON or FO diets differed in the secretory response to a low-grade LPS challenge, as evidenced by decreased IL-6, CCL2, CCL5, and CCL7 secreted from FO ACM (Figure 1). Additionally, in the chronic inflammation model, compared to CON ACM, macrophages treated with FO ACM had decreased mRNA expression of M1-associated markers (*Nos2*, *Tnf*α, *Ccl2*, *Il1*β*, Cd80*, and *Cd86*, Figure 6) and IL-6 and CCL2 secretion (Figure 7); some of these effects were lost when Ad was neutralized, and were further exacerbated when both Ad and LPS were neutralized. Taken together, this work provides evidence that LC n-3 PUFA and Ad may work in concert to suppress specific M1 macrophage responses in a microenvironment representative of AT.

Intriguingly, the results suggest that the anti-inflammatory effects of LC n-3 PUFA in macrophages may be partly due to Ad-mediated signaling, which, to the best of our knowledge, has not been previously reported. Although previous studies have shown that LC n-3 PUFA increase circulating Ad in rodents (43, 44) and humans (45, 46), the current study did not find any changes in secreted levels of full-length Ad in ACM due to diet or LPS exposure (Figure 1B). This may have occurred because the 24 h *ex vivo* period used to generate ACM was not long enough to induce changes in the levels of Ad secreted from the AT organ cultures; however, this requires further study. Nonetheless, some of the anti-inflammatory effects of FO ACM on macrophages in both the acute and chronic inflammation models were lost when Ad was neutralized. For example, in the acute model, lipid uptake increased back to control level in FO ACM-treated macrophages when Ad was neutralized in LPS-challenged cells (Figure 2). Decreasing lipid uptake may be a mechanism by which LC n-3 PUFA act to decrease excessive M1 macrophage responses in obese AT since lipotoxicity in macrophages is thought to be a key driver of M1 macrophage polarization (47). Second, in the chronic inflammation model, compared to LPS control, the FO ACM-mediated decrease in mRNA expression of M1-associated markers (*Nos2*, *Ccl2*, *Nlrp3*, and *Cd80*, Figure 6) and secretion of CCL2 (Figure 7) was lost when Ad was neutralized. Taken together, this data suggest that Ad plays a role in LC n-3 PUFA-mediated anti-inflammatory effects in macrophages, such as buffering lipid uptake and mRNA expression of specific markers associated with the M1 macrophage phenotype.

Another novel finding in the current study was our data showing that Ad may work synergistically with LPS to decrease macrophage mRNA expression of M1 markers, particularly in FO ACM-treated macrophages in the chronic inflammation model. Specifically, M1 markers (*Itgam*, *Il6*, Figures 6A,B), NLPR3 inflammasome genes (*Nlrp3*, *Il18*, *Il1*β, Figure 6C), MHCII-related antigen presentation genes (*Cd74*, *Cd80*, *Cd86*, Figure 6D), as well as secretion of IL-6 and TNFα (Figure 7), further increased compared to FO ACM with anti-Ad when both anti-Ad plus the LPS-neutralizing agent, polymyxin B, were added. Along these lines, Park et al. (18, 48) showed that globular Ad initially triggers signaling cascades that result in activation of NFκB and subsequent TNFα mRNA expression; however, 5 h post-induction, this triggered a compensatory anti-inflammatory response resulting in IL-10 secretion and subsequent desensitization when re-stimulated with LPS. Thus, Ad may have immunoregulating properties in response to an inflammatory stimulus, such as LPS, which warrants greater characterization, particularly in FO ACM-treated macrophages where some of the anti-inflammatory effects of Ad were negated when both Ad and LPS were blocked. Indeed, future studies silencing adiponectin signaling in primary macrophages would be an effective strategy to delineate if adiponectin plays a role in the immunomodulatory activity of LC n-3 PUFA in macrophages.

Interestingly, in the acute inflammation model, neutralizing Ad in both CON and FO ACM led to a decrease in mRNA expression of several M1-associated markers (*Tnf*α, *Il6*, *Ccl5*, Figure 3A; NLRP3 inflammasome genes *Nlrp3*, *Casp1*, *Il18*, Figure 3A; secretion of IL-6 and CCL2, Figure 3B), as well as a decrease in expression of M2 markers (*Il10* and *Cd206*, Figure 4). This data suggest that Ad promotes mRNA expression of both M1 and M2 genes in ACM, which is in line with a previous report showing that M1 macrophages treated with Ad showed more robust inflammatory cytokine secretion (e.g., IL-6), and M2 polarized macrophages treated with Ad showed more robust IL-10 secretion (19). This relationship requires further study *in vivo* especially given that a recent study in obese mice showed Ad accumulation in AT stroma vascular cells, including macrophages (49), further suggesting that Ad could potentially promote more robust M1 and M2 macrophage polarization in obese AT.

Our findings that FO ACM had marked anti-inflammatory effects in macrophages compared to CON ACM in both the acute and chronic inflammation models are in agreement with other studies showing that LC n-3 PUFA decrease the degree of M1 macrophage polarization in the chronically inflamed obese AT microenvironment *in vivo* (26–29, 50). Notably, compared to CON ACM generated from AT organ cultures, FO ACM had decreased secreted protein levels of IL-6, CCL2, CCL5, and CCL7 (Figure 5). Second, compared to CON ACM, FO ACM decreased mRNA expression of M1-associated markers in macrophages, including *Nos2*, *Nf*κ*b*, *Il6*, *Il18*, *Ccl2*, and *Ccl5* in the acute model (Figure 3A). Third, compared to CON ACM, FO ACM decreased mRNA expression of M1 markers (*Nos2*, *Tnf*α, *Ccl2*, *Il18*, *Il1*β, *Cd80*, and *Cd86*, Figure 6) and secretion of IL-6 and CCL2 in the chronic inflammation model (Figure 7), and this occurred concurrently with changes in the macrophage lipid profile. Notably, macrophage LC n-3 PUFA, such as DPA and DHA increased, while SFA, such as stearic acid, decreased (Figure 5). This data show that LC n-3 PUFA are released from AT within ACM and that these fatty acids are subsequently taken up by macrophages. This is not surprising since n-3 PUFA-fed animals and humans exhibit increased AT n-3 PUFA storage and mobilization [reviewed by Raclot (51)]. Additionally, TLR4 stimulation causes DHA and EPA pre-treated macrophages to secrete these PUFA as free fatty acids (52), which represents a potential mechanism by which LC n-3 PUFA distribute themselves among macrophages to mitigate excessive inflammatory responses. Furthermore, this change in macrophage lipid profile could partly explain some of the anti-inflammatory effects observed after treating macrophages with FO ACM. After LC n-3 PUFA are taken up by macrophages, the subsequent anti-inflammatory effects may be partly due to their incorporation into the phospholipid fraction of cellular membranes where they can act to decrease the signaling efficiency of protein complexes localized in small, hydrophobic membrane microdomains called lipid rafts (53, 54), including the TLR4 complex (55); however, this relationship requires further study in ACM models.

Treating macrophages with FO ACM also led to changes in macrophage mRNA expression of MHCII-related antigen presentation genes. Specifically, compared to CON ACM, macrophages treated with FO ACM had decreased mRNA expression of co-stimulatory molecules, including *Cd86* in the acute inflammation model (Figure 4), and *Cd80* and *Cd86* (Figure 6D) in the chronic inflammation model. This data complements previous research showing that LC n-3 PUFA decrease antigen presenting cell-mediated co-stimulatory molecule mRNA and surface expression (56, 57). Moreover, globular (16, 18) and full-length (15, 58–60) Ad promote IL-10 secretion from macrophages. IL-10 represses co-stimulatory molecule expression on antigen presenting cells (61), suggesting that the Ad-IL-10 axis may affect antigen presenting cell functions. In this study, FO ACM-treated macrophages did not upregulate IL-10 mRNA expression or secretion, suggesting that the FO-mediated decrease in mRNA expression of *Cd80* and *Cd86* occurs independently of IL-10 in this model. Overall, this data provide preliminary evidence that FO may mitigate antigen presentation in macrophages [key antigen presenting cells in obese AT (9, 62, 63)] by decreasing co-stimulatory molecule expression; however, this relationship requires further study.

In summary, we showed for the first time that ACM promotes a response similar to endotoxin tolerance in macrophages and that macrophages treated with FO ACM had decreased lipid uptake and expression of markers associated with an inflammatory M1 phenotype compared to CON ACM in both the acute and chronic inflammation models. Importantly, the anti-inflammatory effect of FO ACM on suppressing lipid uptake and M1 marker expression in macrophages was partly lost when Ad was neutralized in FO ACM. Overall, our data suggests that Ad and LC n-3 PUFA work together to affect the macrophage phenotype in a microenvironment representative of obese AT. Additionally, this relationship warrants further study *in vivo* to further understand the role of dietary LC n-3 PUFA in mitigating obese AT inflammation and related pathologies.

## Author Contributions

AD, JM, and LR designed the research; AD, JM, and DL conducted the research; AD analyzed the data; AD and LR wrote the paper and had primary responsibility for the final content; DM and KP provided the animals and expertise. All authors read and approved the final manuscript.

## Conflict of Interest Statement

The authors declare that the research was conducted in the absence of any commercial or financial relationships that could be construed as a potential conflict of interest.

## Funding

This work was supported by the Natural Sciences and Engineering Research Council of Canada (NSERC) and Agriculture and Agri-Food Canada. AB was supported by a College of Biological Sciences PhD award at the University of Guelph and a NSERC PGSD scholarship. DL was supported by an Ontario Graduate Scholarship.

## Abbreviations

ACM, adipose tissue conditioned media; Ad, adiponectin; AT, adipose tissue; ATM, adipose tissue macrophage; CD, cluster of differentiation; CCL, chemokine (c–c motif) ligand; CON, control; DHA, docosahexaenoic acid; EPA, eicosapentaenoic acid; FO, fish oil; IL, interleukin; iNOS, inducible nitric oxide synthase; LC n-3 PUFA, long-chain n-3 polyunsaturated fatty acids; LF, low-fat; LPS, lipopolysaccharide; NFκB, nuclear factor kappa-light-chain-enhancer of activated B cells; NLRP, NOD-like receptor family, Pyrin domain containing; PBS, phosphate buffered saline; Px, polymyxin B; ROS, reactive oxygen species; TLR, toll-like receptor; TNFα, tumor necrosis factor alpha.

## References

[1] Winer S Chan Y Paltser G Truong D Tsui H Bahrami J Normalization of obesity-associated insulin resistance through immunotherapy. Nat Med (2009) 15:921–9. 10.1038/nm.2001 19633657 PMC3063199

[2] Nishimura S Manabe I Nagasaki M Eto K Yamashita H Ohsugi M CD8 effector T cells contribute to macrophage recruitment and adipose tissue inflammation in obesity. Nat Med (2009) 15:914–20. 10.1038/nm.1964 19633658

[3] Kosteli A Sugaru E Haemmerle G Martin JF Lei J Zechner R Weight loss and lipolysis promote a dynamic immune response in murine adipose tissue. J Clin Invest (2010) 120:3466. 10.1172/JCI42845 20877011 PMC2947229

[4] Xu X Grijalva A Skowronski A van Eijk M Serlie MJ Ferrante AW. Obesity activates a program of lysosomal-dependent lipid metabolism in adipose tissue macrophages independently of classic activation. Cell Metab (2013) 18:816–30. 10.1016/j.cmet.2013.11.001 24315368 PMC3939841

[5] Dey A Allen J Hankey-Giblin PA. Ontogeny and polarization of macrophages in inflammation: blood monocytes versus tissue macrophages. Front Immunol (2014) 5:683. 10.3389/fimmu.2014.00683 25657646 PMC4303141

[6] Wentworth JM Naselli G Brown WA Doyle L Phipson B Smyth GK Pro-inflammatory CD11c CD206 adipose tissue macrophages are associated with insulin resistance in human obesity. Diabetes (2010) 59:1648–56. 10.2337/db09-0287 20357360 PMC2889764

[7] Lumeng CN Bodzin JL Saltiel AR. Obesity induces a phenotypic switch in adipose tissue macrophage polarization. J Clin Invest (2007) 117:175. 10.1172/JCI29881 17200717 PMC1716210

[8] Fujisaka S Usui I Bukhari A Ikutani M Oya T Kanatani Y Regulatory mechanisms for adipose tissue M1 and M2 macrophages in diet-induced obese mice. Diabetes (2009) 58:2574–82. 10.2337/db08-1475 19690061 PMC2768159

[9] Morris DL Cho KW DelProposto JL Oatmen KE Geletka LM Martinez-Santibanez G Adipose tissue macrophages function as antigen-presenting cells and regulate adipose tissue CD4 T cells in mice. Diabetes (2013) 62:2762–72. 10.2337/db12-1404 23493569 PMC3717880

[10] Suganami T Nishida J Ogawa Y. A paracrine loop between adipocytes and macrophages aggravates inflammatory changes role of free fatty acids and tumor necrosis factor α. Arterioscler Thromb Vasc Biol (2005) 25:2062–8. 10.1161/01.ATV.0000183883.72263.13 16123319

[11] Cani PD Amar J Iglesias MA Poggi M Knauf C Bastelica D Metabolic endotoxemia initiates obesity and insulin resistance. Diabetes (2007) 56:1761–72. 10.2337/db06-1491 17456850

[12] West MA Heagy W Endotoxin tolerance: a review. Crit Care Med (2002) 30:S64–73. 10.1097/00003246-200201001-00009 11782563

[13] Folco EJ Rocha VZ López-Ilasaca M Libby P. Adiponectin inhibits pro-inflammatory signaling in human macrophages independent of interleukin-10. J Biol Chem (2009) 284:25569–75. 10.1074/jbc.M109.019786 19617629 PMC2757958

[14] Ohashi K Parker JL Ouchi N Higuchi A Vita JA Gokce N Adiponectin promotes macrophage polarization toward an anti-inflammatory phenotype. J Biol Chem (2010) 285:6153–60. 10.1074/jbc.M109.088708 20028977 PMC2825410

[15] Mandal P Pratt BT Barnes M McMullen MR Nagy LE. Molecular mechanism for adiponectin-dependent M2 macrophage polarization link between the metabolic and innate immune activity of full-length adiponectin. J Biol Chem (2011) 286:13460–9. 10.1074/jbc.M110.204644 21357416 PMC3075692

[16] Zacharioudaki V Androulidaki A Arranz A Vrentzos G Margioris AN Tsatsanis C. Adiponectin promotes endotoxin tolerance in macrophages by inducing IRAK-M expression. J Immunol (2009) 182:6444–51. 10.4049/jimmunol.0803694 19414798

[17] Tsatsanis C Zacharioudaki V Androulidaki A Dermitzaki E Charalampopoulos I Minas V Adiponectin induces TNF-α and IL-6 in macrophages and promotes tolerance to itself and other pro-inflammatory stimuli. Biochem Biophys Res Commun (2005) 335:1254–63. 10.1016/j.bbrc.2005.07.197 16115611

[18] Park P McMullen MR Huang H Thakur V Nagy LE. Short-term treatment of RAW264.7 macrophages with adiponectin increases tumor necrosis factor-α (TNF-α) expression via ERK1/2 activation and Egr-1 expression. J Biol Chem (2007) 282:21695. 10.1074/jbc.M701419200 17537727 PMC1978175

[19] Van Stijn CMW Kim J Lusis AJ Barish GD Tangirala RK. Macrophage polarization phenotype regulates adiponectin receptor expression and adiponectin anti-inflammatory response. FASEB J (2014) 29:636–49. 10.1096/fj.14-253831 25392268 PMC4314235

[20] Lovren F Pan Y Quan A Szmitko PE Singh KK Shukla PC Adiponectin primes human monocytes into alternative anti-inflammatory M2 macrophages. Am J Physiol Heart Circ Physiol (2010) 299:H656–63. 10.1152/ajpheart.00115.2010 20622108 PMC2944489

[21] Calder PC. n-3 polyunsaturated fatty acids, inflammation, and inflammatory diseases. Am J Clin Nutr (2006) 83:S1505–19. 16841861 10.1093/ajcn/83.6.1505S

[22] Galli C Calder PC Effects of fat and fatty acid intake on inflammatory and immune responses: a critical review. Ann Nutr Metab (2009) 55:123–39. 10.1159/000228999 19752539

[23] De Boer AA Monk JM Robinson LE. Docosahexaenoic acid decreases pro-inflammatory mediators in an in vitro murine adipocyte macrophage co-culture model. PLoS One (2014) 9:e85037. 10.1371/journal.pone.0085037 24465472 PMC3896343

[24] Oliver E McGillicuddy FC Harford KA Reynolds CM Phillips CM Ferguson JF Docosahexaenoic acid attenuates macrophage-induced inflammation and improves insulin sensitivity in adipocytes-specific differential effects between LC n-3 PUFA. J Nutr Biochem (2012) 23:1192–200. 10.1016/j.jnutbio.2011.06.014 22137266

[25] Awada M Meynier A Soulage CO Hadji L Géloën A Viau M n-3 PUFA added to high-fat diets affect differently adiposity and inflammation when carried by phospholipids or triacylglycerols in mice. Nutr Metab (2013) 10:23. 10.1186/1743-7075-10-23 23413782 PMC3585798

[26] Ludwig T Worsch S Heikenwalder M Daniel H Hauner H Bader BL. Metabolic and immunomodulatory effects of n-3 fatty acids are different in mesenteric and epididymal adipose tissue of diet-induced obese mice. Am J Physiol Endocrinol Metab (2013) 304:E1140–56. 10.1152/ajpendo.00171.2012 23482450

[27] Yan Y Jiang W Spinetti T Tardivel A Castillo R Bourquin C Omega-3 fatty acids prevent inflammation and metabolic disorder through inhibition of NLRP3 inflammasome activation. Immunity (2013) 38:1154–63. 10.1016/j.immuni.2013.05.015 23809162

[28] Monk JM Hou TY Turk HF Weeks B Wu C McMurray DN Dietary n-3 polyunsaturated fatty acids (PUFA) decrease obesity-associated Th17 cell-mediated inflammation during colitis. PLoS One (2012) 7:e49739. 10.1371/journal.pone.0049739 23166761 PMC3500317

[29] Titos E Rius B González-Périz A López-Vicario C Morán-Salvador E Martínez-Clemente M Resolvin D1 and its precursor docosahexaenoic acid promote resolution of adipose tissue inflammation by eliciting macrophage polarization toward an M2-like phenotype. J Immunol (2011) 187:5408–18. 10.4049/jimmunol.1100225 22013115

[30] Oster RT Tishinsky JM Yuan Z Robinson LE. Docosahexaenoic acid increases cellular adiponectin mRNA and secreted adiponectin protein, as well as PPARγ mRNA, in 3T3-L1 adipocytes. Appl Physiol Nutr Metab (2010) 35:783–9. 10.1139/H10-076 21164549

[31] Tishinsky JM Ma DWL Robinson LE. Eicosapentaenoic acid and rosiglitazone increase adiponectin in an additive and PPARγ-dependent manner in human adipocytes. Obesity (2010) 19:262–8. 10.1038/oby.2010.186 20814411

[32] Fried SK Moustaid-Moussa N Culture of adipose tissue and isolated adipocytes. Methods Mol Biol (2001) 155:197–212. 11293072 10.1385/1-59259-231-7:197

[33] Creely SJ McTernan PG Kusminski CM Fisher FM Da Silva NF Khanolkar M Lipopolysaccharide activates an innate immune system response in human adipose tissue in obesity and type 2 diabetes. Am J Physiol Endocrinol Metab (2007) 292:E740–7. 10.1152/ajpendo.00302.2006 17090751

[34] MacLennan MB Clarke SE Perez K Wood GA Muller WJ Kang JX Mammary tumor development is directly inhibited by lifelong n-3 polyunsaturated fatty acids. J Nutr Biochem (2013) 24:388–95. 10.1016/j.jnutbio.2012.08.002 23026490

[35] Fats and Fatty Acid in Human Nutrition. Available from: http://www.fao.org/3/a-i1953e.pdf

[36] Halliwell B Gutteridge JMC. The antioxidants of human extracellular fluids. Arch Biochem Biophys (1990) 280:1–8. 10.1016/0003-9861(90)90510-6 2191627

[37] Ho P-C Wei L-N. Negative regulation of adiponectin secretion by receptor interacting protein 140 (RIP140). Cell Signal (2012) 24:71–6. 10.1016/j.cellsig.2011.07.018 21872658 PMC3205305

[38] Rasouli N Yao-Borengasser A Varma V Spencer HJ McGehee RE Peterson CA Association of scavenger receptors in adipose tissue with insulin resistance in nondiabetic humans. Arterioscler Thromb Vasc Biol (2009) 29:1328–35. 10.1161/ATVBAHA.109.186957 19667111 PMC2755066

[39] Folch J Lees M Sloane-Stanley GH A simple method for the isolation and purification of total lipids from animal tissues. J Biol Chem (1957) 226:497–509. 13428781

[40] Lau B Ward W Kang J Ma D. Femur EPA and DHA are correlated with femur biomechanical strength in young fat-1 mice. J Nutr (2009) 20:453–61. 10.1016/j.jnutbio.2008.05.004 18708283

[41] Green H Kehinde O. An established preadipose cell line and its differentiation in culture II. Factors affecting the adipose conversion. Cell (1975) 5:19–27. 10.1016/0092-8674(75)90087-2 165899

[42] Monk JM Liddle DM De Boer AA Brown MJ Power KA Ma DWL Fish-oil-derived n-3 PUFAs reduce inflammatory and chemotactic adipokine-mediated cross-talk between co-cultured murine splenic CD8 T cells and adipocytes. J Nutr (2015) 145:829–38. 10.3945/jn.114.205443 25833786

[43] Flachs P Mohamed-Ali V Horakova O Rossmeisl M Hosseinzadeh-Attar MJ Hensler M Polyunsaturated fatty acids of marine origin induce adiponectin in mice fed a high-fat diet. Diabetologia (2006) 49:394–7. 10.1007/s00125-005-0053-y 16397791

[44] Tishinsky JM Gulli RA Mullen KL Dyck DJ Robinson LE. Fish oil prevents high-saturated fat diet-induced impairments in adiponectin and insulin response in rodent soleus muscle. Am J Physiol Regul Integr Comp Physiol (2012) 302:R598–605. 10.1152/ajpregu.00328.2011 22204953

[45] Wu JHY Cahill LE Mozaffarian D. Effect of fish oil on circulating adiponectin: a systematic review and meta-analysis of randomized controlled trials. J Clin Endocrinol Metab (2013) 127:2451–9. 10.1210/jc.2012-3899 23703724 PMC3667269

[46] Von Frankenberg AD Silva FM de Almeida JC Piccoli V do Nascimento FV Sost MM Effect of dietary lipids on circulating adiponectin: a systematic review with meta-analysis of randomised controlled trials. Br J Nutr (2014) 112:1235–50. 10.1017/S0007114514002013 25192422

[47] Prieur X Mok CYL Velagapudi VR Núñez V Fuentes L Montaner D Differential lipid partitioning between adipocytes and tissue macrophages modulates macrophage lipotoxicity and M2/M1 polarization in obese mice. Diabetes (2011) 60:797–809. 10.2337/db10-0705 21266330 PMC3046840

[48] Park P Huang H McMullen MR Bryan K Nagy LE. Activation of cyclic-AMP response element binding protein contributes to adiponectin-stimulated interleukin-10 expression in RAW 264.7 macrophages. J Leukoc Biol (2008) 83:1258–66. 10.1189/jlb.0907631 18263767

[49] Nakatsuji H Kishida K Sekimoto R Komura N Kihara S Funahashi T Accumulation of adiponectin in inflamed adipose tissues of obese mice. Metabolism (2014) 63:542–53. 10.1016/j.metabol.2013.12.012 24467915

[50] Todoric J Löffler M Huber J Bilban M Reimers M Kadl A Adipose tissue inflammation induced by high-fat diet in obese diabetic mice is prevented by n-3 polyunsaturated fatty acids. Diabetologia (2006) 49:2109–19. 10.1007/s00125-006-0300-x 16783472

[51] Raclot T. Selective mobilization of fatty acids from adipose tissue triacylglycerols. Prog Lipid Res (2003) 42:257–88. 10.1016/S0163-7827(02)00066-8 12689620

[52] Norris PC Dennis EA. Omega-3 fatty acids cause dramatic changes in TLR4 and purinergic eicosanoid signaling. Proc Natl Acad Sci U S A (2012) 109:8517–22. 10.1073/pnas.1200189109 22586114 PMC3365225

[53] Stulnig TM Huber J Leitinger N Imre E-M Angelisová P Nowotny P Polyunsaturated eicosapentaenoic acid displaces proteins from membrane rafts by altering raft lipid composition. J Biol Chem (2001) 276:37335–40. 10.1074/jbc.M106193200 11489905

[54] Turk HF Chapkin RS. Membrane lipid raft organization is uniquely modified by n-3 polyunsaturated fatty acids. Prostaglandins Leukot Essent Fat Acids (2013) 88(1):43–7. 10.1016/j.plefa.2012.03.008 22515942 PMC3404206

[55] Wong SW Kwon M-J Choi AMK Kim H-P Nakahira K Hwang DH. Fatty acids modulate toll-like receptor 4 activation through regulation of receptor dimerization and recruitment into lipid rafts in a reactive oxygen species-dependent manner. J Biol Chem (2009) 284:27384–92. 10.1074/jbc.M109.044065 19648648 PMC2785667

[56] Weatherill AR Lee JY Zhao L Lemay DG Youn HS Hwang DH. Saturated and polyunsaturated fatty acids reciprocally modulate dendritic cell functions mediated through TLR4. J Immunol (2005) 174:5390–7. 10.4049/jimmunol.174.9.5390 15843537

[57] Schoeniger A Adolph S Fuhrmann H Schumann J. The impact of membrane lipid composition on macrophage activation in the immune defense against *Rhodococcus equi* and *Pseudomonas aeruginosa*. Int J Mol Sci (2011) 12:7510–28. 10.3390/ijms12117510 22174614 PMC3233420

[58] Wolf AM Wolf D Rumpold H Enrich B Tilg H. Adiponectin induces the anti-inflammatory cytokines IL-10 and IL-1RA in human leukocytes. Biochem Biophys Res Commun (2004) 323:630–5. 10.1016/j.bbrc.2004.08.145 15369797

[59] Kumada M Kihara S Ouchi N Kobayashi H Okamoto Y Ohashi K Adiponectin specifically increased tissue inhibitor of metalloproteinase-1 through interleukin-10 expression in human macrophages. Circulation (2004) 109:2046–9. 10.1161/01.CIR.0000127953.98131.ED 15096450

[60] Kollias A Tsiotra PC Ikonomidis I Maratou E Mitrou P Kyriazi E Adiponectin levels and expression of adiponectin receptors in isolated monocytes from overweight patients with coronary artery disease. Cardiovasc Diabetol (2011) 10:14–26. 10.1186/1475-2840-10-14 21284833 PMC3042923

[61] Grutz G. New insights into the molecular mechanism of interleukin-10-mediated immunosuppression. J Leukoc Biol (2005) 77:3–15. 10.1189/jlb.0904484 15522916

[62] Cho KW Morris DL DelProposto JL Geletka L Zamarron B Martinez-Santibanez G An MHC II-dependent activation loop between adipose tissue macrophages and CD4 T cells controls obesity-induced inflammation. Cell Rep (2014) 9:605–17. 10.1016/j.celrep.2014.09.004 25310975 PMC4252867

[63] Dalmas E Venteclef N Caer C Poitou C Cremer I Aron-Wisnewsky J T cell-derived IL-22 amplifies IL-1β-driven inflammation in human adipose tissue: relevance to obesity and type 2 diabetes. Diabetes (2014) 63:1966–77. 10.2337/db13-1511 24520123

